# Efficacy and safety of TACE-HAIC combined with tyrosine kinase inhibitors and immune checkpoint inhibitors for the patients with BCLC-defined stage B-C HCC

**DOI:** 10.3389/fonc.2025.1615506

**Published:** 2025-07-29

**Authors:** Bowen Liu, Linan Yin, Yuxin Chen, Xunbo Hou, Yingchen Li, Xuesong Liu, Ruibao Liu

**Affiliations:** Department of Interventional Radiology, Harbin Medical University Cancer Hospital, Harbin, Heilongjiang, China

**Keywords:** hepatocellular carcinoma, transarterial chemoembolization, hepatic arterial infusion chemotherapy, tyrosine kinase inhibitors, immune checkpoint inhibitors, propensity score matching

## Abstract

**Background:**

To evaluate the therapeutic efficacy and safety profile of combining transarterial chemoembolization (TACE) with hepatic arterial infusion chemotherapy (HAIC) combined with tyrosine kinase inhibitors (TKIs) and immune checkpoint inhibitors (ICIs) in patients with hepatocellular carcinoma (HCC) classified as Barcelona Clinic Liver Cancer (BCLC) stage B or C.

**Methods:**

This single-center retrospective analysis included patients with intermediate-to-advanced HCC diagnosed and treated between January 2020 and December 2023. Of 197 eligible patients meeting inclusion criteria, 103 were allocated to the TACE+HAIC+TKI+ICI (T+H+T+I) group and 94 to the HAIC+TKI+ICI (H+T+I) group. Propensity score matching (PSM) was employed to minimize confounding bias, yielding 50 patients per group in the final matched cohorts. Comparative analyses assessed overall survival (OS), progression-free survival (PFS), objective response rate (ORR), and disease control rate (DCR). Primary endpoints were OS and PFS; secondary endpoints included ORR and safety outcomes.

**Result:**

Among the 100 patients included in the analysis, 50 patients received T+H+T+I therapy while the remaining 50 underwent H+T+I treatment, with median follow-up durations of 13.1 months and 14.3 months, respectively. After PSM, the baseline characteristics showed no significant differences between the two groups. The T+H+T+I group demonstrated superior median overall survival (mOS) (20.77 months [95% CI: 11.37-30.16] vs 14.23 months [95% CI: 12.23-16.24]; *P*=0.019) and longer median progression-free survival (mPFS) (15.43 months [95% CI: 11.85-19.02] vs 10.60 months [95% CI: 7.71-13.49]; *P*<0.001) as assessed by modified Response Evaluation Criteria in Solid Tumors (mRECIST) version 1.1. The T+H+T+I regimen exhibited superior tumor control outcomes, with an ORR of 54% and DCR of 76%. However, this group also showed increased toxicity profiles, with 14 patients (28%) experiencing grade ≥3 adverse events.

**Conclusion:**

For patients with BCLC stage B-C HCC, the T+H+T+I combination therapy demonstrated superior survival benefits, particularly in those with tumor diameter ≥5 cm and presence of portal vein tumor thrombosis (PVTT), while maintaining an acceptable safety profile.

## Introduction

Primary liver cancer ranks as the sixth most common malignancy globally and the third leading cause of cancer-related mortality. HCC constitutes the predominant histopathological subtype, accounting for 75%–85% of cases (1). Early-stage HCC may be managed through surgical resection, ablation therapies, or liver transplantation. However, due to the insidious onset of HCC and stringent eligibility criteria for curative interventions, only 30%–40% of patients ultimately receive definitive treatment at early disease stages (2, 3). In clinical practice, a proportion of patients with intermediate-stage HCC (BCLC-B stage) face unresectable hepatocellular carcinoma (uHCC) due to multifactorial constraints, thereby falling into the same therapeutic category as those diagnosed with advanced-stage disease (BCLC-C stage). For this patient population where curative interventions are not feasible, systemic therapy emerges as an evidence-based treatment strategy (4).

TACE and HAIC remain established locoregional options for uHCC (5). Recent evidence from the LEAP-012 trial indicates that TACE combined with systemic therapy yields improved clinical outcomes (6). In patients with uHCC refractory to multiple TACE sessions, hypoxia induced by repetitive embolization triggers vascular endothelial growth factor (VEGF) upregulation, potentially driving compensatory neovascularization and diminishing TACE efficacy (7, 8). Concurrently, TACE induces immunomodulatory effects within tumor lesions, which may synergistically potentiate the therapeutic efficacy of ICIs. TKIs exhibit dual antiangiogenic activity and immunomodulatory mechanisms that synergize with ICIs (9, 10). For uHCC patients with high tumor burden, conventional TACE often fails to achieve complete embolization due to heterogeneous tumor vasculature. HAIC utilizing oxaliplatin, calcium folinate, and 5-fluorouracil (FOLFOX regimen) delivered via sustained infusion into tumor-feeding arteries prolongs drug exposure, offering an alternative therapeutic strategy (11). Furthermore, HAIC has demonstrated significant survival benefits in HCC patients with PVTT (12, 13). The TRIPLET study further validated the clinical acceptability of HAIC combined with systemic therapy in advanced-stage HCC (14). This retrospective investigation aims to evaluate the efficacy and safety of combined TACE+HAIC with ICI and TKI therapy in BCLC stage B-C HCC patients.

## Methods

### Patients and study design

This retrospective study enrolled 249 patients with intermediate-to-advanced HCC treated at our institution between June 2020 and December 2023, who underwent either TACE combined with HAIC, TKI, and ICI or HAIC with TKI-ICI therapy, with inclusion criteria requiring: (a) age ≥18 years; (b) HCC diagnosis confirmed through clinical history; (c) imaging features, and/or pathological evidence; (d) at least one intrahepatic measurable lesion per mRECIST v1.1; (e) BCLC stage B or C classification; (f) preserved hepatic function (Child-Pugh class A/B); (g) Eastern Cooperative Oncology Group performance status (ECOG PS) 1-2; while exclusion criteria encompassed: (a) prior surgical resection or locoregional therapies (e.g., ablation therapy, TACE); (b) decompensated cirrhosis (Child-Pugh class C, refractory ascites, or overt hepatic encephalopathy); (c) incomplete laboratory/radiological data; (d) concurrent primary malignancies; (e) irregular treatment adherence or loss to follow-up; (f) severe cardiovascular/hematological/renal comorbidities, with all enrolled patients completing at least one cycle of targeted and immunotherapy following TACE+HAIC or HAIC procedures.

### Treatment procedure

All TACE procedures in this study were conventional TACE (c-TACE), utilizing lipiodol and absorbable gelatin sponge as embolic materials. No patient received drug-eluting beads or bland microspheres. Following standard preoperative preparation, the Seldinger technique was utilized for percutaneous femoral artery puncture and sheath insertion. Digital subtraction angiography of the celiac trunk and/or superior mesenteric artery was performed to identify dominant tumor-feeding vessels. A microcatheter was super selectively advanced into the primary feeding artery, followed by administration of a lipiodol-pirarubicin emulsion (lipiodol volume adjusted per lesion size/number, maximum 20 mL; pirarubicin 20–40 mg). After achieving satisfactory oil deposition, the microcatheter remained positioned for subsequent infusion. For tumors demonstrating multiple arterial supplies, staged embolization is systematically performed. Following complete embolization of secondary feeding arteries, supplemental terminal embolization with absorbable gelatin sponge is administered. Ultimately, the microcatheter is left *in situ* within the dominant feeding artery to maintain vascular access for subsequent interventions. Post-procedure, patients received FOLFOX-regimen HAIC via infusion pump: oxaliplatin 100 mg/m² (2-hour infusion), calcium folinate 400 mg/m² (2-hour infusion), and 5-fluorouracil administered as either 2400 mg/m² over 46 hours or 1200 mg/m² over 23 hours, with post-treatment hepatic function reassessment.

The HAIC protocol mirrored TACE-HAIC preparatory steps including angiography and microcatheter positioning, omitting embolization phases. All patients underwent repeat interventions at 3–4 week intervals until disease progression or intolerable toxicity occurred.

### TKIs plus ICIs administration

Systemic targeted and immunotherapeutic agents were initiated within one week post-intervention. The targeted agents included donafenib and lenvatinib, while the immunotherapeutic agents comprised camrelizumab and sintilimab. Specific dosing regimens were as follows: (a) Donafenib: 0.2 g orally twice daily. For grade ≥3 non-hematological or grade 4 hematological adverse events (AEs): First occurrence: Interrupt treatment until resolution to ≤grade 1. If resolved within 1 week: Resume at 0.2 g twice daily; If resolved within 2 weeks: Reduce to 0.2 g once daily; Recurrence after dose reduction: Further reduce to 0.2 g every other day. (b) Lenvatinib: Baseline: 8 mg/day (<60 kg) or 12 mg/day (≥60 kg). For persistent/intolerable grade 2–3 AEs: First occurrence: Interrupt until ≤grade 1, then reduce to 4 mg/day (<60 kg) or 8 mg/day (≥60 kg); Second occurrence: Interrupt until ≤grade 1, then reduce to 4 mg every other day (<60 kg) or 4 mg/day (≥60 kg). (c) Camrelizumab/Sintilimab: 200 mg via intravenous infusion every 3 weeks. Treatment protocols mandated immediate suspension of all therapeutic agents upon development of life-threatening toxicities or severe hepatic impairment (Child-Pugh score deterioration ≥2 points within 2 weeks), accompanied by comprehensive symptomatic supportive care until clinical resolution.

### Follow-up and assessments

All patients underwent scheduled follow-up assessments within 4–8 weeks post-treatment, comprising evaluation of vital signs, clinical symptoms, abdominal contrast-enhanced CT or liver MRI, chest CT, and laboratory investigations (hepatic/renal function, complete blood count, and alpha-fetoprotein [AFP] levels) to monitor treatment response and adverse events. The primary endpoint was OS, defined as the interval from treatment initiation to death from any cause, with censoring at the last follow-up date. Secondary endpoints included PFS – measured from treatment commencement to first radiologic progression or death per mRECIST – ORR, proportion of patients achieving confirmed complete/partial responses by mRECIST, DCR, ORR plus stable disease rat, and safety profiles. Follow-up data were censored on June 30, 2024, with all adverse events systematically recorded from treatment initiation and managed per institutional protocols. Treatment-related adverse events (TRAEs) were graded using the National Cancer Institute Common Terminology Criteria for Adverse Events version 5.0 (NCI-CTCAE v5.0).

### Statistical analysis

All statistical analyses were performed using IBM SPSS Statistics 27 and R 4.4.3. Baseline characteristics were compared using appropriate tests according to variable types: categorical variables were analyzed with Pearson’s χ² test or Fisher’s exact test, while continuous variables underwent normality assessment via the Kolmogorov-Smirnov test, followed by independent samples t-test for normally distributed data or Wilcoxon rank-sum test for nonparametric distributions. PSM was implemented in a 1:1 ratio with a caliper width of 0.02 to balance intergroup covariates. Survival outcomes were estimated using the Kaplan-Meier method and compared through log-rank testing. Cox proportional hazards regression models were employed for subgroup analyses to identify independent prognostic factors associated with OS. All statistical tests were two-sided, with *P* < 0.05 considered statistically significant.

## Results

### Baseline characteristics

**Figure 1:**
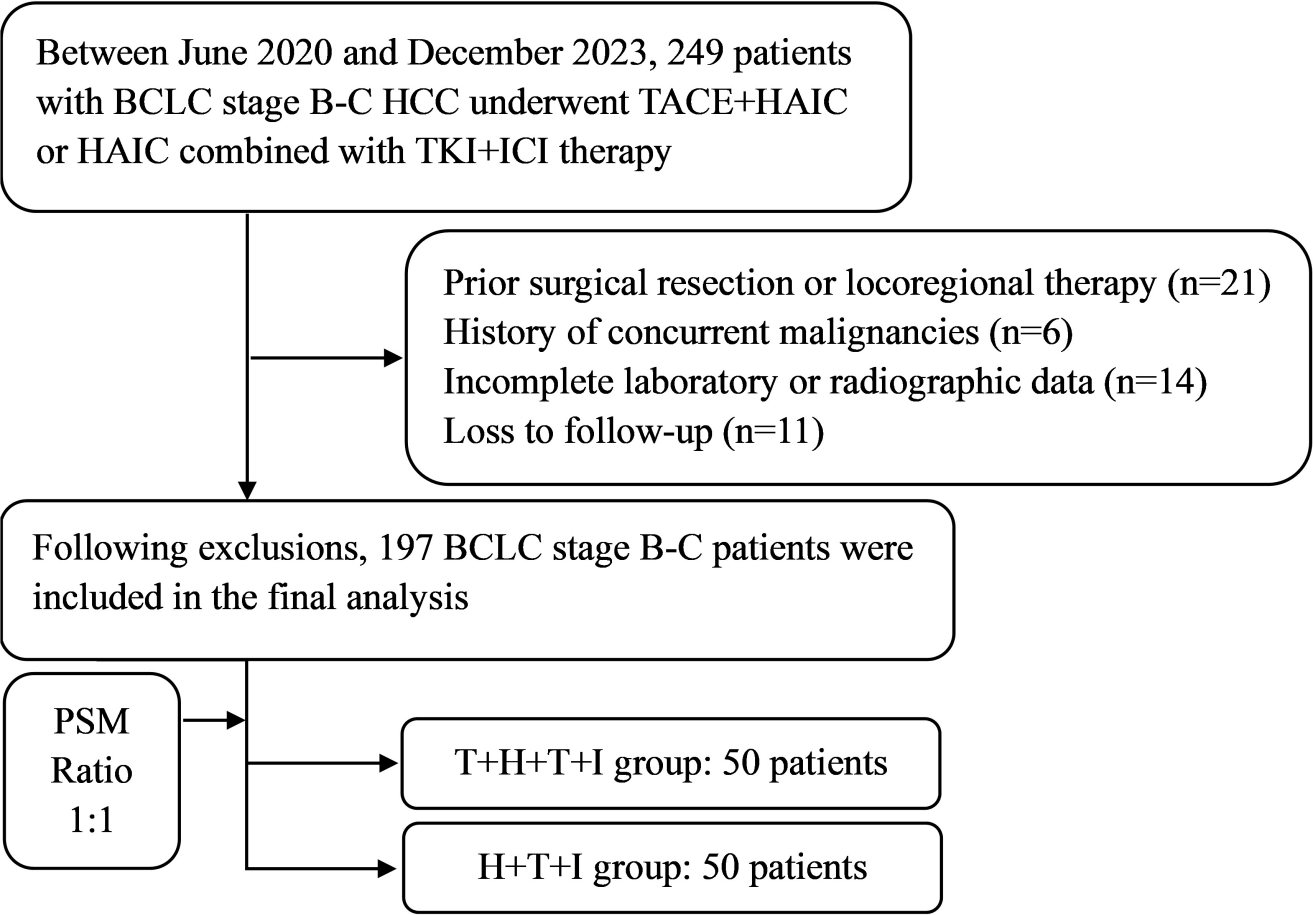
The study flowchart. *BCLC* Barcelona Clinic Liver Cancer, *HCC*, Hepatocellular carcinoma, *T+H+T+I* Transarterial chemoembolization with hepatic arterial infusion chemotherapy combined with tyrosine kinase inhibitor and immune checkpoint inhibitor, *H+T+I*, Hepatic arterial infusion chemotherapy combined with tyrosine kinase inhibitor and immune checkpoint inhibitor.

Following screening protocols, the study population comprised 103 patients allocated to the T+H+T+I group and 94 patients in the H+T+I group. Baseline characteristics (**Table 1**) revealed intergroup differences pre-PSM: compared with the H+T+I group, the T+H+T+I cohort had a higher proportion of patients with PVTT (76 [73.8%] vs 57 [60.6%], *P*=0.049), elevated median serum albumin levels (38.70 g/L [IQR 34.10–41.90] vs 36.80 g/L [IQR 33.80–40.45], *P*=0.040), and increased international normalized ratio (INR) values (1.06 [IQR 1.00–1.13] vs 1.09 [IQR 1.02–1.18], *P*=0.045). Following 1:1 PSM with a 0.02 caliper, both groups demonstrated well-balanced baseline profiles, resulting in 50 matched patients per group. The study flowchart is presented in **Figure 1**.

**Table 1:** Baseline characteristics of the patients.

Variables	Before matching		p value	After matching		p value
	T+H+T+I(n=103)	H+T+I (n=94)		T+H+T+I (n=50)	H+T+I (n=50)	
Sex			0.335			1.000
Male	86 (83.5)	83 (88.3)		43 (86.0)	43 (86.0)	
Female	17 (16.5)	11 (11.7)		7 (14.0)	7 (14.0)	
ECOG PS score			0.700			0.812
1	81 (78.6)	76 (80.6)		38 (76.0)	39 (78.0)	
2	22 (21.4)	18 (19.1)		12 (24.0)	11 (22.0)	
Child-Pugh class			0.878			1.000
A	82 (79.6)	74 (78.7)		40 (80.0)	40 (80.0)	
B	21 (20.4)	20 (21.3)		10 (20.0)	10 (20.0)	
BCLC stage			0.081			0.617
B	16 (15.5)	24 (25.5)		9 (18.0)	11 (22.0)	
C	87 (84.5)	70 (74.5)		41 (82.0)	39 (78.0)	
Hepatitis			0.802			0.799
Yes	90 (87.4)	81 (86.2)		41 (82.0)	40 (80.0)	
No	13 (12.6)	13 (13.8)		9 (18.0)	10 (20.0)	
Cirrhosis			0.111			0.779
Yes	81 (78.6)	82 (87.2)		43 (86.0)	42 (84.0)	
No	22 (21.4)	12 (12.8)		7 (14.0)	8 (16.0)	
Solitary tumor			0.553			0.523
Yes	37 (35.9)	30 (31.9)		15 (30.0)	18 (36.0)	
No	66 (64.1)	64 (68.1)		35 (70.0)	32 (64.0)	
Largest tumor size (cm)			0.483			0.401
<5	18 (17.5)	13 (13.8)		6 (40.0)	9 (18.0)	
≥5	85 (82.5)	81 (86.2)		44 (88.0)	41 (82.0)	
PVTT			0.049			1.000
Yes	76 (73.8)	57 (60.6)		34 (68.0)	34 (68.0)	
No	27 (26.2)	37 (39.4)		16 (32.0)	16 (32.0)	
VP classification			0.180			0.753
Vp0	26 (25.2)	37 (39.4)		16 (32.0)	16 (32.0)	
Vp2	3 (2.9)	1 (1.1)		0 (0.0)	1 (2.0)	
Vp3	47 (45.6)	32 (34.0)		18 (36.0)	19 (38.0)	
Vp4	27 (26.2)	24 (25.5)		16 (32.0)	14 (28.0)	
Extrahepatic metastasis			0.878			0.629
Yes	21 (20.4)	20 (21.3)		38 (76.0)	40 (80.0)	
No	82 (79.6)	74 (78.7)		12 (24.0)	10 (20.0)	
ALBI grade			0.077			0.705
1	34 (33.0)	23 (24.5)		13 (26.0)	12 (24.0)	
2	67 (65.0)	64 (68.1)		36 (72.0)	36 (72.0)	
3	2 (1.9)	7 (7.4)		1 (2.0)	2 (4.0)	
AFP level (ng/ml)			0.739			0.545
≤400	36 (35.0)	35 (37.2)		20 (40.0)	23 (46.0)	
>400	67 (65.0)	59 (62.8)		30 (60.0)	27 (54.0)	
Age	55 (49, 60)	56 (50, 61)	0.192	57 (53, 63)	56 (50, 61)	0.565
BMI (kg/m²)	23.4 (21.5,25.3)	23.9 (21.8, 26.2)	0.327	23.2 (21.6, 25.1)	23.9 (21.9, 26.0)	0.448
ALT (U/l)	39.0 (25.0, 59.0)	34.5 (25.8, 55.0)	0.447	41.0 (27.50, 61.25)	38.0 (27.75, 56.25)	0.728
AST (U/l)	58.0 (37.0, 89.0)	58.5 (39.0, 86.3)	0.771	62.0 (40.5, 88.3)	62.0 (43.5, 95.0)	0.764
γ-GGT (U/l)	171.0 (96.0, 289.0)	180.0 (107.8, 297.3)	0.655	206.5 (114.3, 369.5)	188.5 (118.5, 295.8)	0.600
ALP (U/l)	150.0 (119.0, 211.0)	146.0 (113.8, 204.0)	0.407	166.0 (129.0, 224.5)	150.5 (113.5, 218.3)	0.389
TBIL (umol/l)	20.3 (14.5, 25.7)	21.6 (15.6, 28.7)	0.401	23.2 (17.5, 28.8)	21.1 (14.9, 29.5)	0.567
ALB (g/l)	38.7 (34.1, 41.9)	36.8 (33.8, 40.5)	**0.040**	37.8 (32.8, 41.3)	37.0 (33.8, 40.4)	0.732
PALB (mg/l)	156.0 (110.0, 205.0)	147.5 (104.0, 206.3)	0.433	148.0 (102.5, 199.5)	154.5 (108.0, 208.0)	0.914
Urea (mmol/l)	5.1 (3.9, 6.5)	5.5 (4.2, 6.9)	0.289	5.4 (4.2, 6.4)	4.9 (4.0, 6.8)	0.655
Cr (umol/l)	74.0 (65.0, 86.2)	71.0 (65.0, 80.3)	0.288	73.3 (63.3, 86.7)	70.4 (65.0, 80.0)	0.365
PLT (10^9/l)	165.0 (118.0, 230.0)	155.0 (113.0, 225.5)	0.418	154.5 (104.0, 207.5)	161.5 (112.8, 237.0)	0.512
PT (s)	12.1 (11.6, 13.0)	12.3 (11.7, 13.2)	0.102	12.3 (11.7, 13.1)	12.3 (11.6, 13.2)	0.886
INR	1.06 (1.00, 1.13)	1.09 (1.02, 1.18)	**0.045**	1.07 (1.02, 1.14)	1.08 (1.01, 1.16)	0.438

Values are presented as n (%) or median (Q1, Q3). Bold values denote statistically detectable differences. ALBI (log₁₀ bilirubin × 0.66) + (−0.085 × albumin), BMI weight [kg]/height [m]^2^, PVTT portal vein tumor throm-bosis, ALT Alanine Aminotransferase, AST Aspartate Aminotransferase, γ-GGT Gamma-Glutamyl Transferase, ALP Alkaline Phosphatase, TBIL Total Bilirubin, ALB Serum Albumin, PALB Prealbumin, Urea Blood Urea Nitrogen, Cr Creatinine; PLT Platelet Count, PT Prothrombin Time, INR International Normalized Ratio.

Before PSM treatment cycles totaled 475 TACE-HAIC sessions (range 2–12; median 4) in the T+H+T+I group versus 441 TACE sessions (range 2–11; median 4) in controls. After PSM, intervention cycles decreased to 227 TACE-HAIC procedures (range 2–12; median 4) and 249 TACE sessions (range 2–10; median 5) in the respective groups.

### Efficacy

**Figure 2:**
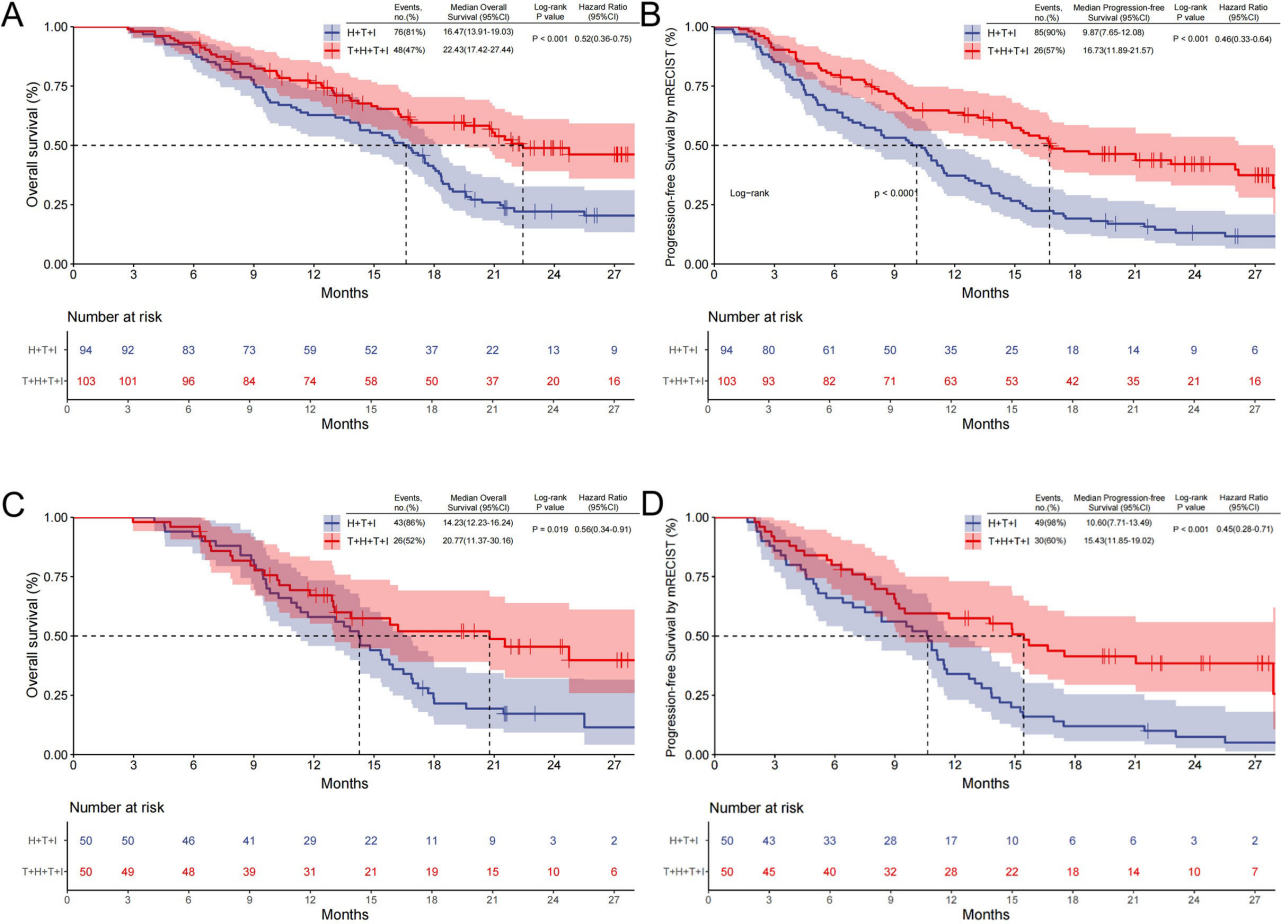
Kaplan–Meier survival curves comparing OS and PFS. **(A)** OS and **(B)** PFS assessed by mRECIST v1.1 (Kaplan-Meier curves before PSM); **(C, D)** analyses after PSM. *T+H+T+I* Transarterial chemoembolization with hepatic arterial infusion chemotherapy combined with tyrosine kinase inhibitor and immune checkpoint inhibitor, *H+T+I*, Hepatic arterial infusion chemotherapy combined with tyrosine kinase inhibitor and immune checkpoint inhibitor; *OS* Overall survival, *PFS* Progression-free survival, *PSM* Propensity score matching.

The T+H+T+I cohort demonstrated a median follow-up of 13.1 months, compared with 14.3 months in the H+T+I group. Before PSM, 48 patients (47%) in the T+H+T+I group experienced mortality, with a mOS of 22.43 months (95% CI: 17.42–27.44), versus 76 deaths (81%) and mOS of 16.47 months (95% CI: 13.91–19.03) in the H+T+I group (*P*<0.001; HR 0.52, 95% CI: 0.36–0.75; **Figure 2A**). mPFS was 16.73 months (95% CI: 11.89–21.57) in the T+H+T+I group versus 9.87 months (95% CI: 7.65–12.08) in controls (*P*<0.001; HR 0.46, 95% CI: 0.33–0.64; **Figure 2B**).

After PSM, 26 deaths (52%) occurred in the T+H+T+I cohort with mOS of 20.77 months (95% CI: 11.37–30.16), contrasting with 43 deaths (86%) and mOS of 14.23 months (95% CI: 12.23–16.24) in the H+T+I group (*P*=0.019; HR 0.56, 95% CI: 0.34–0.91; **Figure 2C**). The T+H+T+I regimen also showed superior mPFS (15.43 months, 95% CI: 11.85–19.02) versus 10.60 months (95% CI: 7.71–13.49) in the comparator group (*P*<0.001; HR 0.45, 95% CI: 0.28–0.71; **Figure 2D**).

**Figure 3:**
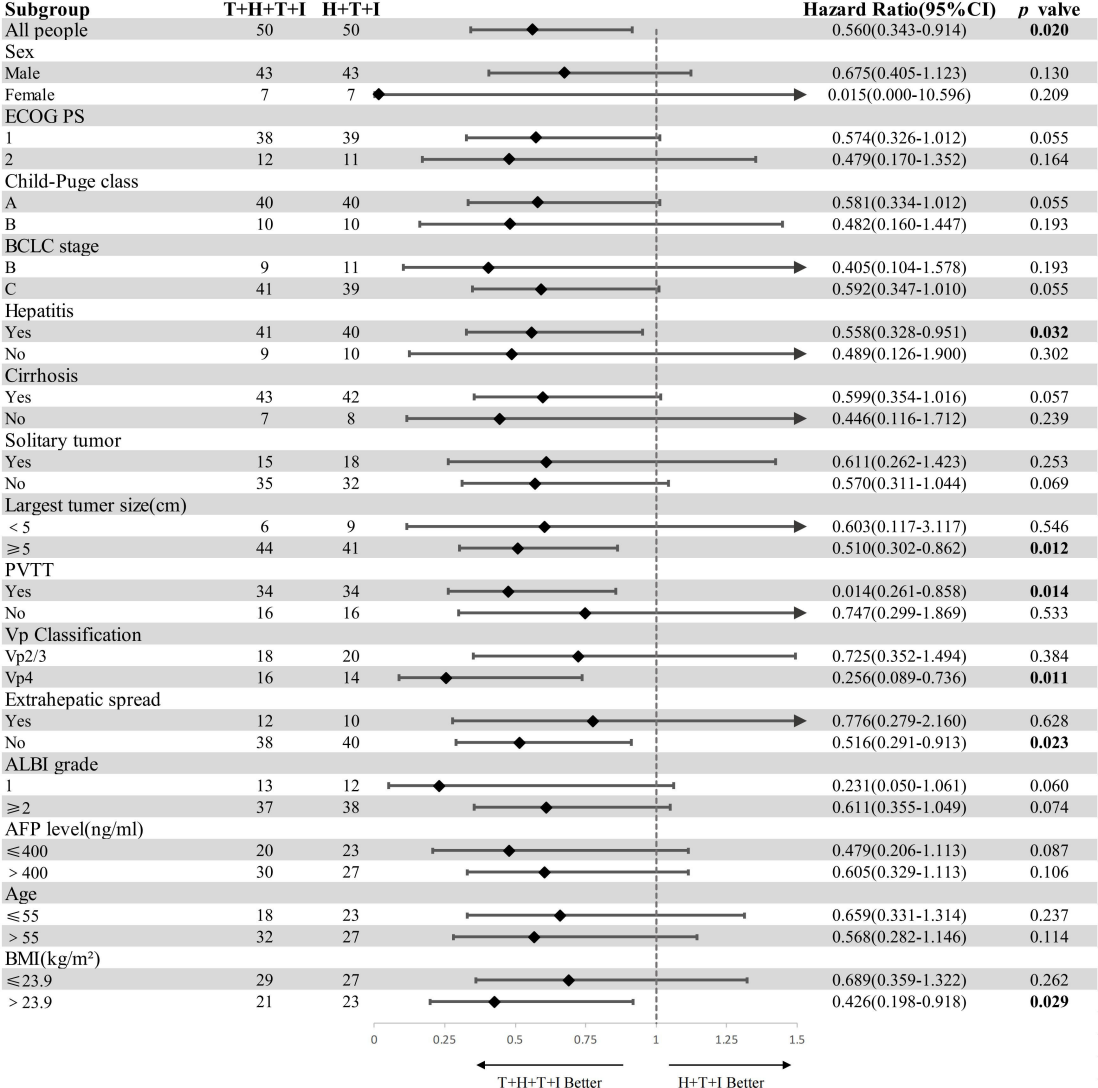
Subgroup analysis of overall survival (forest plot). Bold values denote statistically detectable differences. *HR* hazard ratio, *CI* confidence interval, *ECOG PS* Eastern Cooperative Oncology Group performance status, *BCLC* Barcelona Clinic Liver Cancer, *ALBI* grade (log_10_ bilirubin × 0.66) + (−0.085 × albumin), *AFP* alpha-fetoprotein, *BMI* body mass index = weight/height², *T+H+T+I* transarterial chemoembolization with hepatic arterial infusion chemotherapy combined with tyrosine kinase inhibitor and immune checkpoint inhibitor, *H+T+I* hepatic arterial infusion chemotherapy combined with tyrosine kinase inhibitor and immune checkpoint inhibitor.

Prespecified subgroups were stratified by sex, ECOG PS score, Child-Pugh class, BCLC stage, hepatitis status, cirrhosis, tumor focality (solitary/multiple), maximum diameter, portal vein invasion, VP-classified tumor thrombosis, extrahepatic metastasis, Albumin-Bilirubin (ALBI) score, AFP level, age, and BMI. Forest plot analysis demonstrated superior survival benefit with the T+H+T+I regimen versus H+T+I in the following subgroups: hepatitis-positive patients (*P*=0.032; HR 0.56, 95% CI: 0.328–0.951), tumor diameter ≥5 cm (*P*=0.012; HR 0.49, 95% CI: 0.302–0.862), presence of PVTT (*P*=0.014; HR 0.48, 95% CI: 0.261–0.858), VP-4 tumor thrombosis (*P*=0.011; HR 0.31, 95% CI: 0.089–0.736), absence of distant metastasis (*P*=0.023; HR 0.52, 95% CI: 0.291–0.913), and BMI >23.9 (*P*=0.029; HR 0.46, 95% CI: 0.198–0.918). The survival advantage was particularly pronounced in patients with tumors ≥5 cm, PVTT, or VP-4 thrombus classification (**Figure 3**).

Before PSM, ORR by Response Criteria (RECIST) criteria were comparable between the T+H+T+I group and the H+T+I group (32% vs 30%, *P*=0.733), with similar DCR (73% vs 67%, *P*=0.375). When assessed by mRECIST, the T+H+T+I group showed numerically higher ORR (50% vs 41%) and DCR (76% vs 70%) compared to the H+T+I group, though these differences lacked statistical significance (**Table 2**).

**Table 2:** Before PSM tumor responses.

Characteristic	RECIST (version 1.1)			mRECIST (version 1.1)		
	T+H+T+I	H+T+I	*p* value	T+H+T+I	H+T+I	*p* value
ORR	33 (32%)	28 (30%)	0.733	52 (50%)	39 (41%)	0.206
DCR	75 (73%)	63 (67%)	0.375	78 (76%)	66 (70%)	0.383
CR	3 (3%)	1 (1%)	0.358	11 (11%)	8 (9%)	0.606
PR	30 (29%)	27 (29%)	0.950	41 (40%)	31 (33%)	0.588
SD	42 (41%)	35 (37%)	0.611	26 (25%)	27 (29%)	0.582
PD	28 (27%)	31 (33%)	0.375	25 (24%)	28 (30%)	0.383

*p*-values were calculated using the χ² test. *RECIST* Response Criteria, *mRECIST* modified Response Evaluation Criteria in Solid Tumors. *ORR* Objective Response Rate, *DCR* Disease Control Rate, *CR* Complete Response, PR Partial Response, *SD* Stable Disease, *PD* Progressive Disease. *PSM* Propensity score matching. *T+H+T+I* transarterial chemoembolization with hepatic arterial infusion chemotherapy combined with tyrosine kinase inhibitor and immune checkpoint inhibitor; *H+T+I* hepatic arterial infusion chemotherapy combined with tyrosine kinase inhibitor and immune checkpoint inhibitor.

After PSM, tumor response outcomes remained similar between the T+H+T+I group and the H+T+I group by RECIST (ORR: 34% vs 30%, *P*=0.688; DCR: 70% vs 60%, *P*=0.295; **Table 3**). While mRECIST-based analyses suggested modest advantages for the T+H+T+I group in ORR (54% vs 40%, *P*=0.161) and DCR (76% vs 64%, *P*=0.190) compared to the H+T+I group, no statistically significant intergroup differences were observed across either evaluation framework. Collectively, the T+H+T+I group demonstrated a trend toward improved tumor response metrics compared to the H+T+I group, though without achieving statistical superiority.

**Table 3:** After PSM tumor responses.

Characteristic	RECIST (version 1.1)			mRECIST (version 1.1)		
	T+H+T+I	H+T+I	*p* value	T+H+T+I	H+T+I	*p* value
ORR	17 (34%)	15 (30%)	0.688	27 (54%)	20 (40%)	0.161
DCR	35 (70%)	30 (60%)	0.295	38 (76%)	32 (64%)	0.190
CR	0	0	–	3 (6%)	1 (2%)	0.307
PR	17 (34%)	15 (30%)	0.688	24 (48%)	19 (38%)	0.313
SD	18 (36%)	15 (30%)	0.523	11 (22%)	12 (24%)	0.812
PD	15 (30%)	20 (40%)	0.295	12 (24%)	18 (36%)	0.190

p -values were calculated using the ² test. RECIST Response Criteria, mRECIST modified Response Evaluation Criteria in Solid Tumors. ORR Objective Response Rate, DCR Disease Control Rate, CR Complete Response, PR Partial Response, SD Stable Disease, PD Progressive Disease. PSM Propensity score matching. T+H+T+I transarterial chemoembolization with hepatic arterial infusion chemotherapy combined with tyrosine kinase inhibitor and immune checkpoint inhibitor; H+T+I hepatic arterial infusion chemotherapy combined with tyrosine kinase inhibitor and immune checkpoint inhibitor

### Safety

The T+H+T+I group exhibited TRAEs including elevated aspartate aminotransferase (AST) (40%), abdominal pain (38%), thrombocytopenia (36%), and hypertension (34%), while the H+T+I cohort reported elevated AST (36%), abdominal pain (34%), hand-foot syndrome (34%), and thrombocytopenia (32%). Grade ≥3 AEs occurred slightly more frequently in the T+H+T+I group (28% vs 22%, *P*=0.694). Treatment modifications (dose reduction or temporary interruption) due to intolerable toxicities were required in 14 patients (28%) in the T+H+T+I group versus 10 patients (20%) in the comparator group. As detailed in **Table 4**, the T+H+T+I regimen demonstrated a numerically higher incidence of overall AEs; however, its safety profile remained manageable within clinically acceptable parameters.

**Table 4:** Treatment-related adverse events.

Adverse events	T+H+T+I		H+T+I	
	Any Grade	Grade≥3	Any Grade	Grade≥3
Hypertension	17 (34%)	7 (14%)	15 (30%)	5 (10%)
Hypoalbuminemia	8 (16%)	3 (6%)	10 (20%)	1 (2%)
Thrombocytopenia	18 (36%)	4 (8%)	16 (32%)	3 (6%)
Elevated AST	20 (40%)	6 (12%)	18 (36%)	4 (8%)
Elevated TB	12 (24%)	3 (6%)	9 (18%)	1 (2%)
Weight decrease	8 (16%)	0	11 (22%)	0
Diarrhea	17 (34%)	3 (6%)	14 (28%)	3 (6%)
Rash	12 (24%)	1 (2%)	10 (20%)	2 (4%)
Hand-foot syndrome	16 (32%)	2 (4%)	17 (34%)	2 (4%)
Pain abdominal	19 (38%)	5 (10%)	17 (34%)	3 (6%)
Gingival bleeding	3 (6%)	0	5 (10%)	0
Proteinuria	2 (4%)	1 (2%)	4 (8%)	0

*AST*, Aspartate aminotransferase; *TB*, Total bilirubin; *T+H+T+I*, transarterial chemoembolization with hepatic arterial infusion chemotherapy combined with tyrosine kinase inhibitor and immune checkpoint inhibitor; *H+T+I*, hepatic arterial infusion chemotherapy combined with tyrosine kinase inhibitor and immune checkpoint inhibitor.

## Discussion

This retrospective study demonstrated that the T+H+T+I regimen conferred improved OS and PFS compared with the H+T+I regimen. Higher survival rates were observed in subgroups with a tumor diameter ≥5 cm, PVTT, or VP-4 thrombus classification, despite comparable tumor response rates between the two regimens.

The strengths of this study are threefold. This study compares the efficacy between combination therapeutic regimens, a less explored area in current clinical research. While existing studies predominantly focus on evaluating combination therapies (TACE+TKI+ICI or HAIC+TKI+ICI) against monotherapies (e.g., TACE alone), limited evidence exists regarding head-to-head comparisons of distinct combination strategies. Second, the inclusion of a real-world, heterogeneous uHCC population—encompassing multifocal tumors, high tumor burden, main portal trunk/first-branch tumor thrombosis, and extrahepatic metastasis—ensures clinical relevance and informs personalized therapeutic strategies for advanced HCC. Third, methodologically, we implemented refined 1:1 PSM across demographic characteristics, tumor profiles, baseline hepatic/renal function, and nutritional/coagulation parameters, systematically mitigating confounding biases and enhancing result reliability.

For the results obtained in this study, we will conduct the following analysis: the current real-world study CHANCE-2201 suggests that TACE combined with systemic therapy is superior to TACE treatment alone (15); The median OS reached 22.6 months. Meanwhile, in the subgroup analysis of OS in CHANCE-2201, combination therapy was found to be superior to TACE alone in subgroups with HBV infection, large vessel invasion, and no extrahepatic metastasis. The forest plot of subgroup analysis in this study suggests that only in some subgroups, the survival benefit of the T+H+T+I group is better than that of the H+T+I group, especially in subgroups with tumor diameter ≥ 5CM, presence of portal vein cancer thrombus and VP4 type cancer thrombus. However, in other subgroups, there is no significant statistical difference because this study compared the efficacy of combination therapy and did not show similar results in terms of survival benefit as other studies.

Notably, the median PFS of 10.60 months in our H+T+I group exceeds the 9.53 months reported by Zhang et al. (14) for HAIC combined with camrelizumab plus apatinib. Similarly, Yuan et al. (16) observed a median PFS of 14.8 months with combination therapy versus TACE alone in PVTT-positive HCC, slightly lower than our T+H+T+I cohort’s 15.43 months. This discrepancy may reflect our inclusion of BCLC stage B patients, potentially contributing to marginally improved survival metrics. However, the lack of OS benefit in BCLC B subgroups—likely attributable to limited sample size—warrants cautious interpretation. Furthermore, a meta-analysis by Haber et al. (17) suggested that ICI therapy demonstrated enhanced efficacy in patients with virus-related hepatitis-induced HCC, whereas TKI efficacy showed no significant association with etiology. This may partially account for the superior survival benefits observed with the T+H+T+I combination regimen in the hepatitis-positive subgroup.

TACE induces the release of tumor antigens and proinflammatory cytokines, and locally triggers immunogenic cell death in HCC to further enhance immune responses (18); The chemotherapeutic agents in HAIC modulate the immune microenvironment by enhancing CD8+ T cell infiltration into tumors, which may synergize with ICIs to achieve better outcomes (19, 20);Lu et al. (21) performed a meta-analysis comparing HAIC combined with systemic therapy versus systemic therapy alone, indicating that the integration of HAIC with systemic treatment confers favorable survival benefits in advanced HCC. Both TACE and HAIC elicit antitumor immune responses through microenvironment remodeling, thereby synergizing with immunotherapy. Hypoxia induced by TACE promotes angiogenesis; TKIs counter this effect through their multi-kinase inhibitory activity, which confers anti-proliferative and anti-angiogenic properties (22);Fan et al.’s (23) randomized clinical trial demonstrated that for patients with recurrent intermediate-stage HCC and microvascular invasion (MVI) positivity after hepatectomy, TACE combined with sorafenib was superior to TACE alone. Pro-angiogenic factors such as VEGF are frequently upregulated in HCC patients. VEGF impairs leukocyte-endothelial interactions, limiting immune cell infiltration. Consequently, VEGF blockade may increase effector T-cell populations and potentiate immunotherapy (10);Zhang et al.’s meta-analysis revealed that the TACE plus lenvatinib and ICIs group demonstrated superior efficacy compared to the TACE plus lenvatinib group, with an acceptable safety profile. HAIC demonstrates superior efficacy in patients with large tumors or portal vein invasion. In contrast, TACE monotherapy often fails to achieve complete tumor devascularization in high-burden HCC, adjunctive administration of particulate embolic agents not only fails to confer prognostic improvement (24), but also elevates risks of non-target embolization and hepatic decompensation. Studies by Deng et al. (25) and He et al. (26) indicate that HAIC achieves superior tumor control rates compared to TACE in patients with massive HCC lacking vascular invasion or distant metastasis, HAIC also provides higher intratumoral drug concentration, reduced systemic toxicity. Patients with PVTT, particularly VP3/4 subtypes, exhibit poorer prognosis (27), and are ineligible for surgical resection. While combined local and systemic therapies are essential, no standardized treatment protocols exist for this subgroup. Local treatment options include radioactive seed implantation (28) and radiotherapy (29). TACE monotherapy shows limited efficacy in these patients, with studies reporting increased ischemic liver injury risk in PVTT cases, especially those with compromised hepatic reserve or insufficient portal collateral circulation (30). HAIC is prioritized as a treatment option in Japan and Taiwan (31, 32). Hu et al. (33) demonstrated superior survival benefits of HAIC over TACE/TAE in HCC patients with PVTT. Thus, HAIC addresses the limitations of TACE monotherapy in managing large tumors and PVTT, which may explain the improved OS and PFS observed in the T+H+T+I cohort of this study.

The T+H+T+I cohort demonstrated marginally superior tumor response rates compared to the H+T+I group. In this trial, the tumor response rate of the T+H+T+I cohort was comparable to that of the TACE combined with sorafenib plus ICIS regimen in Zheng et al.’s study (34) (ORR: 54.0% vs. 54.5%). Potential explanations include this study’s larger sample size and poorer baseline characteristics (particularly tumor size) in the enrolled population. Zhou et al. (35) reported a 6-month DCR (61.4% vs. 64%) similar to this study in their HAIC combined with lenvatinib and immune checkpoint inhibitor cohort, though with a higher ORR. This discrepancy may stem from differences in evaluation timing and the relatively smaller sample size of the H+T+I subgroup. No statistically significant differences in tumor response were observed between groups either before or after PSM. This may reflect the comprehensive baseline heterogeneity in the study population. Subgroup stratification based on tumor burden or vascular invasion patterns might reveal statistically significant positive outcomes regarding tumor control.

Regarding safety profiles, the T+H+T+I cohort exhibited a marginally higher incidence of AEs. Treatment-related pain and hepatic impairment were primarily attributable to TACE and HAIC procedures. Compared to HAIC monotherapy, the combined TACE+HAIC approach imposed greater hepatic burden and resulted in a higher incidence of postprocedural pain. Cutaneous reactions (rash, hand-foot syndrome) and gingival bleeding occurred at comparable frequencies in both cohorts, likely owing to their shared systemic therapy components. Notably, the incidence of grade ≥3 AEs showed no statistically significant intergroup difference, aligning with historical data from prior combination therapy studies, thus supporting the acceptable safety profile of the T+H+T+I regimen.

This retrospective study has several inherent limitations. Although PSM mitigated baseline disparities, residual selection bias persists. The single-center design constrained post-matching sample sizes, particularly in subgroup analyses, resulting in inconclusive findings for certain subgroups. Furthermore, single-center data limit generalizability due to the absence of multicenter validation with standardized protocols. To address these limitations, multicenter prospective trials are warranted. Such trials should implement stratified designs based on treatment center, hepatic function, and vascular invasion status, while establishing standardized certification systems for interventional procedures. Uniform imaging assessment criteria and quality control of therapeutic processes should be implemented to ensure sufficient subgroup power and robust outcome evaluation for validating the efficacy and safety of combination therapies in BCLC stage B-C patients. Additionally, this study did not utilize drug-eluting beads as embolic materials; future investigations could conduct subgroup analyses regarding embolic agent selection.

## Conclusion

The TACE+HAIC+TKI+ICI combination therapy demonstrates prolonged survival, favorable tumor response rates, and acceptable safety-particularly in patients with tumor diameter ≥5 cm or PVTT. This multimodal regimen represents a potential therapeutic option for advanced HCC with high tumor burden or vascular invasion, pending validation in prospective multicenter trials.

## Data Availability

The original contributions presented in the study are included in the article/supplementary material. Further inquiries can be directed to the corresponding author.
